# Plant senescence and proteolysis: two processes with one destiny

**DOI:** 10.1590/1678-4685-GMB-2016-0015

**Published:** 2016-08-08

**Authors:** Mercedes Diaz-Mendoza, Blanca Velasco-Arroyo, M. Estrella Santamaria, Pablo González-Melendi, Manuel Martinez, Isabel Diaz

**Affiliations:** Centro de Biotecnología y Genómica de Plantas, Universidad Politécnica de Madrid, Madrid, Spain

**Keywords:** barley, cysteine-proteases, leaf senescence, protein traffic, protein recycling, proteolysis

## Abstract

Senescence-associated proteolysis in plants is a complex and controlled process, essential for mobilization of nutrients from old or stressed tissues, mainly leaves, to growing or sink organs. Protein breakdown in senescing leaves involves many plastidial and nuclear proteases, regulators, different subcellular locations and dynamic protein traffic to ensure the complete transformation of proteins of high molecular weight into transportable and useful hydrolysed products. Protease activities are strictly regulated by specific inhibitors and through the activation of zymogens to develop their proteolytic activity at the right place and at the proper time. All these events associated with senescence have deep effects on the relocation of nutrients and as a consequence, on grain quality and crop yield. Thus, it can be considered that nutrient recycling is the common destiny of two processes, plant senescence and, proteolysis. This review article covers the most recent findings about leaf senescence features mediated by abiotic and biotic stresses as well as the participants and steps required in this physiological process, paying special attention to C1A cysteine proteases, their specific inhibitors, known as cystatins, and their potential targets, particularly the chloroplastic proteins as source for nitrogen recycling.

## Proteolysis is associated with leaf senescence

Leaf senescence is a physiological process critical for plant survival. It is characterized by the dismantling of cellular structures, massive degradation of macromolecules and efficient relocation of nutrients from senescing leaves to growing tissues or sink organs (Gregersen *et al.*, 2008, Krupinska *et al.*, 2012, Avice and Etiene, 2014, Diaz-Mendoza *et al.*, 2014). This coordinated sequence of events associated with senescence is triggered by the reprograming of thousands of genes, down- or up-regulated, in response to specific senescence-promoting factors. Accordingly, many hydrolytic enzymes targeted to degrade proteins, lipids, nucleic acids and pigments are activated. At the same time, basic metabolic activities are maintained to ensure the processing of high molecular weight molecules and the subsequent mobilization of the hydrolyzed products to the phloem (Gregersen *et al.*, 2008, Yang and Ohlrogge, 2009, Roberts *et al.*, 2012, Avila-Ospina *et al.*, 2014, Sakamoto and Takami, 2014).

**Figure 1:**
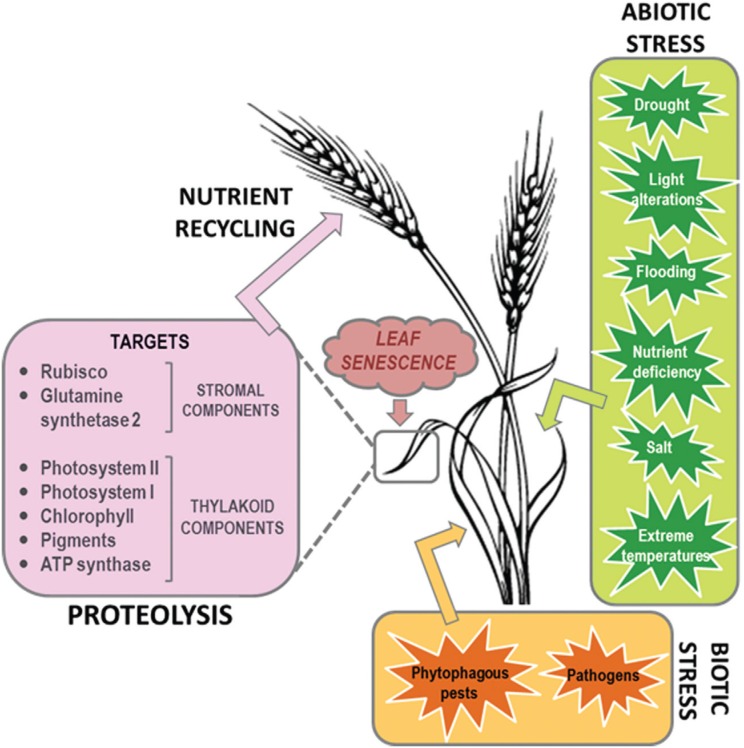
Physiological events involved in induced-senescence mediated by biotic/abiotic stresses. Multiple biotic and abiotic stresses induce leaf senescence characterized by a dismantling of organelles and proteolysis, mainly from chloroplastic proteins. In consequence, protein breakdown and mobilization from stressed tissues to growing and sink organs are the major metabolic features essential for nutrient recycling.

Protein breakdown is one of the most important catabolic processes associated with leaf senescence with an essential role in nutrient recycling, especially nitrogen. Changes in the temporal expression pattern of proteases take place not only in nuclei but also in chloroplasts and mitochondria to cooperatively ensure protein degradation into amino acids, amides and ammonium (Diaz-Mendoza *et al.*, 2014, Roberts *et al.*, 2012). As a result, a complex traffic of proteins, peptides and amino acids takes place among cell compartments involving chloroplasts, cytosol, special vesicles and lytic vacuoles (Roberts *et al.*, 2012, Carrion *et al.*, 2013, Avila-Ospina *et al.*, 2014, Diaz-Mendoza *et al.*, 2014). Finally, the major part of the nitrogen is released as ammonium after being re-assimilated into amino acids to be exported via the phloem to developing grains, fruits and tubers. In consequence, the timing of leaf senescence is of pivotal importance for yield in crop species (Gregersen *et al.*, 2013). Figure 1 summarizes the whole set of events related to the proteolytic processes during leaf senescence.

## Leaf senescence is induced by abiotic and biotic stresses

Leaf senescence is a natural developmental process but it is also closely linked to abiotic and biotic stresses. This physiological set of events can be modulated by endogenous and exogenous factors such as plant growth regulators (abscisic acid, cytokinin, ethylene, salicylic acid), sucrose starvation, dark, cold, heat, drought, salt, or wound. Moreover, pathogen infection (bacteria, fungi, viruses) and phytophagous arthropod infestation can also promote or alter senescence (Figure 1). There are numerous reports demonstrating how abiotic stresses trigger leaf senescence by reprograming specific subsets of senescence-associated genes (SAGs) that are differentially expressed in distinct tissues (Roberts *et al.*, 2012, Diaz-Mendoza *et al.*, 2014). This has been reviewed in recent special issues published: J Exp Bot vol. 65 and J Plant Growth Reg vol. 33 in 2014, and Plants vol. 4 in 2015, as well as in other reviews from previous years (Quirino *et al.*, 2000; Yoshida, 2003; Guo and Gan, 2005; Gregersen *et al.*, 2008; Martinez *et al.*, 2008b). In contrast, information about the interplay between leaf senescence and biotic stresses is more limited, particularly with respect to leaf senescence linked to phytophagous pests. Regarding this interaction between senescence and biotic stress, it is sometimes difficult to elucidate which event comes first. Pathogen and pest lifestyles determine the developmental program of the host, and on the other side, the developmental status of the host may affect the outcome of the host-pathogen/pest interactions (Haffner *et al.*, 2015). Pathogen infection and herbivore infestation influence leaf senescence via modulation of the plant metabolite status directly affecting primary metabolism or by regulating levels of plant hormones (Masclaux-Daubresse *et al.*, 2010; Machado *et al.*, 2013; Seifi *et al.*, 2013, Fagard *et al.*, 2014).

There are a wealth of data analysing the relationship between pathogens and plants. Likewise, induced-senescence genes have been detected during the hypersensitive response (HR) against incompatible bacteria and fungi as well as interactions with viruses (Pontier *et al.*, 1999, Schenk *et al.*, 2005, Espinoza *et al.*, 2007, Fernandez-Calvino *et al.*, 2015). The same SAGs were overexpressed during HR produced by fungal, bacterial and viral infection (Fagard *et al.*, 2014). In Arabidopsis and grapevine, transcripts coding for aspartyl- and cysteine-protease (CysProt) increased during senescence and as a part of plant responses during compatible viral interactions (Espinoza *et al.*, 2007). In tobacco, expression of the CysProt *SAG12* was also induced during the HR against viruses and bacteria (Pontier *et al.*, 1999). Down-regulation of *OsSAG12-1* in rice brings about early senescence and enhances cell death when inoculated with *Xanthomonas oryzae* (Singh *et al.*, 2013). Biotic stresses mediated by pathogens induce N mobilization in Arabidopsis (Masclaux-Daubresse *et al.*, 2010, Fagard *et al.*, 2014). However, references about proteolysis in leaf senescence upon arthropod feeding are occasional. Very recently, Kempema *et al.* (2015) have demonstrated that three SAGs, one of them *SAG12*, were induced in Arabidopsis plants by infestation of the hemipteran *Bemisia tabaci*. The green peach aphid, *Myzus persicae*, when fed on Arabidopsis, induces the expression of *SAG13, SAG21* and *SAG27* genes, cell death alongside chlorophyll degradation (Pegadaraju *et al.*, 2005). Petrova and Smith (2015) demonstrated that the application of salivary secretions of the planthopper *Nilaparvata lugens* to rice induced host mRNAs associated with nutrient mobilization.

Plant responses to confluent abiotic and biotic stresses are not only the addition of the responses to independent stress. Abiotic stress factors alter not only plant defence responses but also their susceptibility to biotic interactions (Prasch and Sonnewald, 2013). The presence of an abiotic stress may reduce or enhance susceptibility to a biotic pest or pathogen and *vice versa* (Atkinson and Urwin, 2012). Thus, dark-induced senescence in potato promoted feeding and nymph development of the aphid *Myzus persicae* probably due to amino acid mobilization and phloem sap loading (Machado-Assefh *et al.*, 2014). Similarly, nitrogen deficiency in barley seedlings induced molecular and metabolic adjustments that trigger aphid resistance. This is because N-deficient leaves were enriched in amino acids and sugars providing a more nutritive diet to phloem-feeding insects (Comadira *et al.*, 2015), The metabolic plant profiles demonstrated that plants were adapted to low N availability by reducing photosynthesis but not respiration or protein turnover. The significance of this overlap and the precise roles of biotic- and senescence-responsive pathways remain still unknown.

## Interplay between proteases, protease inhibitors and target proteins

### Proteases

**Figure 2:**
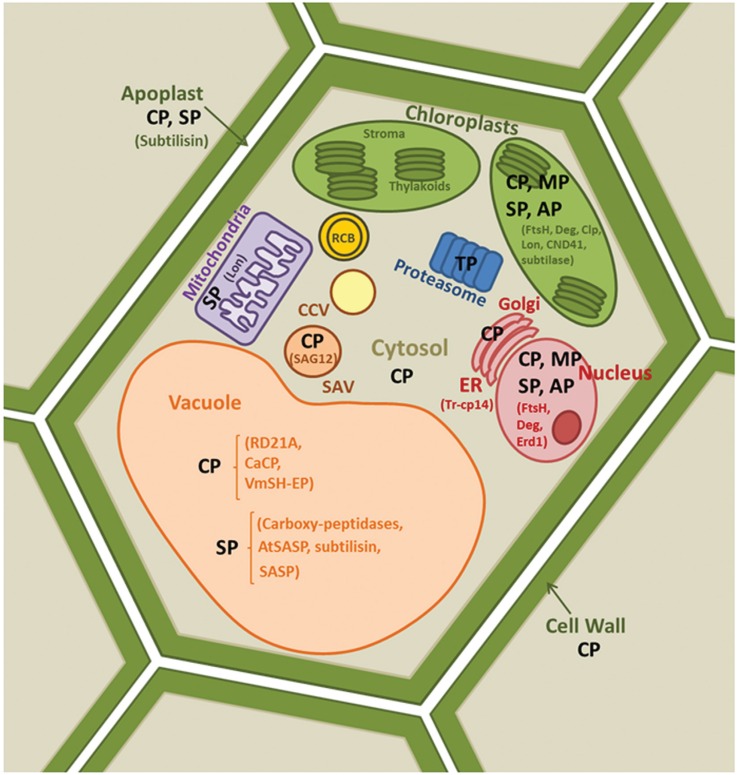
Location of plant proteases in different cellular compartments involved in plant senescence. Different families of proteases are represented: Cysteine-Proteases (CP), Serine-Proteases (SP), Metallo-Proteases (MP), Threonine-Proteases (TP) and Aspartic-Proteases (AP). Subcellular localization of specific proteases, indicated in brackets, are in chloroplast (FtsH-, Clp-, Lon-, DegP- CND41- and subtilase-like proteases), vacuole (RD21A, CaCP, VmSh-EP, AtSAP, subtilase-, SASP-like proteases), mitochondria (Lon-like protease), SAV (SAG12-like protease), ER (Tr-cp-14-like protease) and apoplast (subtilisin). Endoplasmic Reticulum (ER), Rubisco Containing Body (RCB), Senescence Associated Vesicle (SAV) and Chloroplast Containing Vesicles (CCV).

Among the more than 800 proteases identified in plant genomes (Rawlings *et al.*, 2016), serine-proteases and CysProt have been described as the most abundant enzymes associated with leaf senescence in different plant species (Roberts *et al.*, 2012, Diaz and Martinez, 2013, Bhalerao *et al.*, 2003, Diaz-Mendoza *et al.*, 2014, Kidric *et al.*, 2014a). Aspartic-, threonine- and metallo-proteases also participate in this physiological process but their role has been less documented (Graham *et al.*, 1991, Roberts *et al.*, 2012). Expression studies have shown changes in the temporal patterns and subcellular location of proteases during senescence, which is consistent with alterations in proteolytic activities (Breeze *et al.*, 2011, Roberts *et al.*, 2012, Kidric *et al.*, 2014a). Plant proteases have been detected in different cellular compartments such as nuclei, chloroplasts, cytosol, endoplasmic reticulum (ER), vacuoles, mitochondria, apoplast, cell wall or special vesicles (Figure 2), where they fulfil specific functions.

The main proteolytic system in the cytosol is the ubiquitin/26S proteasome pathway, a complex structure involving several proteolytic activities as well as a large set of enzymes needed for covalent binding of targeted proteins to ubiquitin for degradation (Vierstra, 2009). Organelles such as mitochondria, peroxisomes and chloroplasts possess their own conserved proteolytic machinery. In particular, the degradation of the chloroplastic proteins associated with senescence is mediated by the combination of their own proteases and the action of nuclear encoded proteases. These nuclear genes encode precursor proteins with N-terminal extensions known as signal peptides that redirect the processed protein to specific cell locations (Teixeira and Glaser, 2013). Intra-plastidial proteolysis takes place mainly by the action of different forms of FtsH metallo-proteases, Clp serine-proteases, and Lon-like ATP-dependent proteases and DegP serine-proteases, ATP-independent proteases. Members of the DegP, Clp and FtsH proteases are up-regulated in senescing leaves and participate in the degradation of plastidial photosystem II (Roberts *et al.*, 2012). Kato *et al.* (2004) described the proteolytic action of the chloroplast CND41 aspartic-protease on Rubisco (Ribulose 1,5- bisphosphate carboxylase-oxygenase) breakdown during senescence as well as its implication in nitrogen translocation. The over-expression of *CND41* reduced Rubisco in senescent tobacco leaves whereas *CND41*-silenced lines delayed senescence and maintained higher levels of Rubisco in old leaves (Kato *et al.*, 2005). Besides, activities of other chloroplast proteases have been shown to increase under senescence, as in the case of an alkaline serine-protease (subtilase) induced in leaves of spinach under salinity stress and in desiccated leaves of *Ramonda serbica* (Srivastava *et al.*, 2009, Kidric *et al.*, 2014b).

Plastidial proteolysis proceeds outside the organelle through the action of proteases in the cytosol, apoplast or vacuoles (Figure 2). Subunits of the proteasome system are up-regulated by abiotic stress-induced senescence in leaves of tomato and seedlings of *Arabidopsis thaliana* under iron or potassium deficiency (Kidric *et al.*, 2014a). Another example is the Tr-cp14 CysProt of *Trifolium repens*, localized in the ER and associated with senescence in leaves (Mulisch *et al.*, 2013). Proteolytic activities have been also detected in cell walls and inter-cellular spaces (Brzin and Kidric, 1995). Extra-cellular proteases that catalyze the hydrolysis of proteins into peptides and amino acids for subsequent incorporation into the cell constitute a very important step in nitrogen metabolism at this level (Vierstra, 1996, Lopez-Otin and Bond, 2008, Kidric *et al.*, 2014a). Proteolysis during senescence is completed in the acidic environment of the vacuole, which mainly contains C1A CysProt with acidic pH optima, among other enzymes (Thoenen *et al.*, 2007, Ishida *et al.*, 2008, van Doorn *et al.*, 2011). One example is CysProt RD21A, a major protease activity in Arabidopsis leaf extracts and responsible for inducing proteome degradation in the vacuoles of senescing leaves (Yamada *et al.*, 2001, Gu *et al.*, 2012). The silencing of the *CaCP* gene encoding the vacuolar CysProt CaCP of *Capsicum annuum* L. delays salt- and osmotic-induced leaf senescence (Xiao *et al.*, 2014). Although less abundant, carboxy-proteases belonging to serine-protease are also present in vacuoles (van der Hoorn, 2008). For instance, the Arabidopsis AtSASP subtilisin serine-protease has been detected in the proteome of central vacuoles isolated from vegetative leaves (Carter *et al.*, 2004), as well as other two subtilisins, also termed Senescence-Associated Subtilisin Proteases (SASP) with increased proteolytic activity in senescing leaves of this model plant species (Martinez *et al.*, 2015). Recently, Distelfeld *et al.* (2014) have listed several direct and indirect lines of evidence demonstrating the importance of the vacuolar proteases for the complete plastidial protein degradation.

This extra-plastidial pathway of degradation is dependent on ATG genes which contribute at different levels in the autophagy pathway and requires a complex trafficking of proteins from the chloroplast to the central vacuole. A recent review published by Carrion *et al.* (2014) has characterized the Senescence-Associated Vacuoles (SAVs) as specific lytic compartments for degradation of chloroplastic proteins. SAVs coexist with the central vacuole in senescent leaves and they are part of the vesicular transport system where proteolysis may continue due to the presence of active CysProt (Otegui *et al.*, 2005, Martinez *et al.*, 2007, 2008a). The detection of the large subunit of Rubisco and glutamine synthetase 2 (GS2) in a SAV- enriched fraction purified from senescent leaves, and the presence of the CysProt SAG12-GFP, confirms the co-location of both plastidial proteins and CysProt to SAVs during senescence (Martinez *et al.*, 2008a, Carrion *et al.*, 2013). Martinez *et al.* (2008a) also detected a small fraction of chlorophyll *a* using HPLC technology in these acidic vesicles suggesting that pigment disassembly may be carried out through this transport pathway under certain conditions. Moreover, *in vivo* inhibition of CysProt completely abolished Rubisco degradation in isolated SAVs (Carrion *et al.*, 2013, 2014). In addition to SAVs, autophagic bodies named Rubisco-Containing Bodies (RCBs), double membrane bounded vesicles of small size derived from chloroplasts, have been detected in the cytosol of senescent leaves and are also redirected to the central vacuole (Chiba *et al.*, 2003, Prins *et al.*, 2008,). These vesicles carry stromal proteins or their hydrolytic products but not thylakoid proteins, with no evidences of any protease activity within them (Ishida *et al.*, 2008, Carrion *et al.*, 2013). Wang and Blumwald (2014) described a third pathway for the degradation of chloroplastic proteins in stress-induced senescing Arabidopsis based on chloroplast vesiculation (CV), independent of RCBs or SAVs. CCVs (Chloroplast-Containing Vesicles) carry stromal proteins as thylakoid membrane protein (FtsH1), luminal protein (PsbO1) and inner envelope membrane protein (Tic20-II), are released from chloroplasts and redirected to the central vacuole for proteolysis. Further studies are needed to identify partners and protein trafficking routes to senescence in which plastids, vesicles, vacuoles and cytosol are inter-connected for an efficient protein degradation and the subsequent relocation of nutrients.

### Protease activity regulation

Senescence-associated proteolysis in plants is a controlled process where protease activities can be regulated by controlling the protease transcript content through transcriptional regulation, or by control of the activity itself by post-translational processing (Cambra *et al.*, 2012b, Christiansen and Gregersen, 2014, Hollmann *et al.*, 2014). Additionally, enzyme activity is regulated by specific inhibitors and cofactors and through the activation of zymogens. In particular, members of the papain-like subfamily C1A CysProt, probably the most widely studied among plant proteases, are synthesized as inactive or little active precursors to prevent spatio-temporal inappropriate proteolysis. To become active, the C1A proteases are either self-processed or require the aid of other enzymes. Activation takes places by limited intra- or inter-molecular proteolysis cleaving off the inhibitory pro-peptide (Wiederanders, 2003). Therefore, the pro-sequences play important roles as modulators of the protease activity to guarantee that the mature enzyme is formed in the right place and/or at the right time. Pro-peptides are not only able to inhibit their cognate enzymes but also other related proteases *in trans* (Cambra *et al.*, 2012a). Until now, no information has been published about the pro-peptide function as modulators of leaf senescence, but there are some examples demonstrating their regulatory role of such peptides in barley grain germination (Cambra *et al.*, 2012a, 2012b).

Although protease inhibitors act as modulators of the protease activities to control protein turnover, plant protease-inhibitor interactions in response to abiotic stresses are still poorly documented apart from the CysProt and their specific inhibitors known as phytocystatins (Kidric *et al.*, 2014a, Kunert *et al.*, 2015). Cystatins and proteases not only co-localize to the ER and to the Golgi complex, but also interact in these compartments (Martinez *et al.*, 2009). Evidence of *in vivo* interactions has been obtained from BiFC (Bimolecular Fluorescent Complementation) assays using barley cystatins and cathepsin L-like CysProt fused to moieties of the green fluorescent protein marker (GFP). The formation of a CysProt-cystatin complex has also been reported in senescent spinach leaves (Tajima *et al.*, 2011). In a recent publication, Kunert *et al.* (2015) have comented on a current study that they are doing on the interaction between purified recombinant cystatins and CysProt that are expressed during drought using *in vitro* assay systems. These data, in combination with immuno-histochemistry assays will allow them to analyze the intra-cellular localization under optimal and stress conditions. These studies can be essential to demonstrate the specificity of any protease-inhibitor interaction. The CysProt-cystatin interaction has also been indirectly explored in transgenic plants. Prins *et al.* (2008) found an increase of immunogold-labelled Rubisco in chloroplasts as well as in RCBs of tobacco plants overexpressing the rice cystatin OC-I in comparison to the non-transformed controls, whereas OC-I in the cytosol, vacuole, and chloroplasts of these transgenic plants (Prins *et al.*, 2008). Expression of this rice cystatin in soybean and Arabidopsis plants leads to enhanced drought stress tolerance through effects on strigolactone pathways and can also result in improved seed traits (Quain *et al.*, 2014). Overexpression of the broccoli *BoCPI-1* cystatin leads to a decrease in total protease activity and delays chlorophyll degradation and, in consequence, the onset of senescence in broccoli florets after harvest (Eason *et al.*, 2014). Je *et al.* (2014) have shown that DREB2 (Dehydration-Responsive Element-Binding factor) acts as transcriptional activator of the thermotolerance-related *cystatin 4* gene from Arabidopsis, reducing CysProt activity. These findings demonstrate that cystatins can be applied as important regulatory proteins of senescence in biotechnological systems. Other classes of protease inhibitors, mainly targeting serine-proteases, also enhance tolerance to abiotic stress conditions (Shan *et al.*, 2008, Srinivasan *et al.*, 2009), but there is still little information about their importance for leaf senescence.

### Protease targets

Leaf senescence is characterized by a yellowish phenotype parallel to strong plastid disorganization while the rest of the cell organelles remain practically intact until the end of the senescence period. This main hallmark of senescence, leaf yellowing, is caused by the preferential degradation of chlorophyll over carotenoids (Matile, 1992). Chlorophyll degradation occurs by a specific catalytic route termed PAO (from pheophorbide *a* oxygenase). It includes the formation of a primary fluorescent catabolite in the plastid, followed by isomerization to produce non-fluorescent catabolites in the central vacuole (Hörtensteiner, 2013, Christ and Hörtensteiner, 2014). Our own results have demonstrated that a retarded chlorophyll loss parallel to higher protein content is produced in knock-down *HvPap-1* CysProt barley lines grown under darkness (unpublished data). Proteolysis associated with senescence provides free peptides or amino acids and redistributes them within the plant. Degradation of plastidial proteins represents the main source of nitrogen remobilization, and most studies have been focused in this organelle rather than in the other cell compartments (Schiltz *et al.*, 2004, Keech *et al.*, 2007, Avila-Ospina *et al.*, 2014). Most evidence suggests that degradation of stromal proteins, mainly Rubisco and GS2, occurs earlier than degradation of chlorophyll and thylakoidal proteins proteins such as D1, LHCII of the PSII reaction centre and PSII antenna (Krupinska, 2007). However, variability in protein degradation depending on species/cultivars and environmental conditions has also been found (Simova-Stoilova *et al.*, 2010; Krupinska *et al.*, 2012). Degradation of thylakoidal proteins, in particular these from PSII, represents the second largest pool of remobilizable nitrogen from chloroplast during leaf senescence, about 30% of the total chloroplast protein (Matile, 1992; Simova-Stoilova *et al.*, 2010). A faster decline in PSII *vs* PSI was detected during heat stress-promoted leaf senescence in wheat (Hortensteiner and Matile, 2004). Apart from membrane disassembling, photosynthetic proteins both PSI and PSII as well as ATP synthase are hydrolyzed as observed in ultrastructural studies (Ghosh *et al.*, 2001, Guiamet *et al.*, 2002, Krupinska *et al.*, 2012). Immunoblot and ultrastructural results have shown a preferential degradation of granal over stromal proteins, resulting in an unexpected increase in the chlorophyll *a/b* ratio, meaning that chlorophyll *b* is degraded faster (Desimone *et al.*, 1996).

Regarding stromal proteins, Rubisco and Rubisco activase seem to be principal targets for CysProt during leaf senescence in C3 plants (Prins *et al.*, 2008). For this reason, understanding the mechanisms of Rubisco degradation has become a key purpose (Desimone *et al.*, 1996, Schiltz *et al.*, 2004, Irving and Robinson, 2006). Rubisco fragmentation has been detected in intact isolated chloroplasts of pea and wheat incubated under continuous light or dark conditions (Mitsuhashi and Feller, 192, Zhang *et al.*, 2007). Likewise, Desimone *et al.* (1996) studied the nature of Rubisco degradation under oxidative stress in isolated chloroplasts of barley. Several hypotheses put forward that Reactive Oxygen Species (ROS) might be involved in the initial denaturation of Rubisco, by oxydazing certain cysteine residues and thus rendering the protein as a more susceptible target for protease cleavage (Garcia-Ferris and Moreno, 1994). Nonetheless, this ROS prompted degradation does not seem to be sufficient for complete degradation (Desimone *et al.*, 1996). It remains to be elucidated if denaturation and breakdown events are sequential or if both occur at the same time.

GS2 is also susceptible to proteolysis as shown in isolated tobacco chloroplasts. This enzyme is almost lost during early stages of senescence in cereal leaves but the cytosolic GS1, the key enzyme for ammonia assimilation and *de novo* synthesis of amino acids from released nutrients, is maintained (Mitsuhashi and Feller, 1992, Khanna-Chopra, 2012). There are some *in vitro* approaches showing that GS2 is degraded before other enzymes involved in carbon assimilation, such as Rubisco (Thoenen and Feller, 1998). Proteolysis of GS2 seems to be initiated through oxidative carbonylation of histidine residues (Palatnik *et al.*, 1999, Ishida *et al.*, 2002). The presence of only stromal proteins (Rubisco and GS2) within specialized SAVs enriched in CysProt activity, suggests that these enzymes are responsible for the degradation of this stromal protein fraction (Martinez *et al.*, 2008a, Carrion *et al.*, 2013, 2014). Fischer and Feller (1994) examined a broad set of other stromal enzymes in young winter wheat leaves, detecting different degradation rates and discussed how proteolysis within the same organelle can be selectively regulated.

## Influence of leaf senescence on cereal grain yield and quality

Up to 90% of the nitrogen in the grain of several cereals like barley, wheat and rice comes from senescing vegetative tissues. Thomas and Stoddart (1980) postulated that delayed senescence, which means an extended period of maximal photosynthetic activity, should lead to higher yields. However, grain yield is a complex trait that involves different physiological processes. For example, in cereal crops, it is determined by both the source tissues and the sink organs. Fischer (2008) proposed that in small-grain cereals such as wheat, the main limiting factor for grain yield is the strength of the seed, suggesting that the physiological events in the period around seed setting are crucial for determining yield levels. Conversely, there are numerous examples for a positive correlation between delayed senescence and yield levels in both small-grain cereals such as wheat and barley and large-grain cereals such as maize (Gregersen *et al.*, 2013). Furthermore, Egli (2011) stated that enhanced seed yields are obtained when the grain filling period is longer. Thus, manipulation of senescence events could be a way to obtain higher grain yield and quality.

Yield is not the only grain feature affected by plant senescence. Grain quality parameters such as protein and micronutrient (Fe, Zn) concentrations are also affected by alterations in the senescence process (Distelfeld *et al.*, 2014). Grain yield and protein content are negatively correlated in wheat and barley (Thoenen and Feller, 1998, Palatnik *et al.*, 1999, Gregersen *et al.*, 2008). While delayed senescence may lead to higher grain yield but it also provokes inefficient nitrogen remobilization and lower harvest index (Gong *et al.*, 2005), acceleration of senescence confers efficient nitrogen remobilization and high protein content, but also a lower total grain yield. Distelfeld *et al.* (2014) indicated two possible justifications for this effect. The first explanation suggests that in delayed senescence the prolonged accumulation of carbohydrates dilutes stored proteins and micronutrients leading to increased grain weight and yield but lower grain quality (Slafer *et al.*, 1990, Gregersen *et al.*, 2008). The second explanation is based on the fact that the synthesis of storage proteins requires more carbon consumption than the synthesis of starch, which enhances the accumulation of carbohydrates (Munier-Jolain and Salon, 2005).

To cope with this dilemma it is important to balance the agronomical or economic consequences with the quality of the end-product. For barley grains, low protein content is desirable in malting processes to obtain beer whereas high protein content is desirable to be used for animal feeding. Moreover, slow grain filling associated with delayed senescence implies an increased probability of damage by environmental factors such as heat and drought stress and drought during the later stages of crop development (Mi *et al.*, 2002, Yang and Zhang, 2006). Manipulation of the proteolytic machinery is a potential way to enhance grain yield and quality. Using this approach, novel breeding strategies that consider the complexity of the feature are promising tools to achieve a higher grain yield and quality.

## Concluding remarks

Strong indications from multiple studies indicate that prevention of premature senescence induced by biotic/abiotic stresses may be the key in engineering stress tolerance. This review compiled the current knowledge on different aspects related to degradation of proteins as part of the leaf senescence mediated by different stresses, and their potential effects on crop yields. Future research should be done to acquire more precise information about protease action and protease targets and to increase knowledge concerning the traffic of hydrolysed proteins from their original subcellular locations via specialized vesicles to the central lytic vacuole. Clarifying how protease activity is regulated by a specific inhibitor may contribute to understand the balance between recovery from stress and excessive protein degradation resulting in cell death. Overall, research is needed to encompass the complete set of information in order to understand why, how, where and when leaf senescence is produced. The final goal will be to control the impact of senescence in agriculture keeping in mind the current and future effects of extreme weather events related to climate changes.
